# Epidemiology and clinical characteristics of patients with carbapenem-resistant enterobacterales infections: experience from a large tertiary care center in a developing country

**DOI:** 10.1186/s12879-023-08643-9

**Published:** 2023-10-02

**Authors:** Banan M. Aiesh, Yazan Maali, Farah Qandeel, Siwar Omarya, Shatha Abu Taha, Suha Sholi, Ali Sabateen, Adham Abu Taha, Sa’ed H. Zyoud

**Affiliations:** 1 Department of Infection Prevention and Control, An-Najah National University Hospital, Nablus, 44839 Palestine; 2 Department of Medicine, College of Medicine and Health Sciences, An-Najah National University, Nablus, 44839 Palestine; 3 Department of General Surgery, An-Najah National University Hospital, Nablus, 44839 Palestine; 4 Department of Biomedical Sciences, Faculty of Medicine and Health Sciences, An-Najah National University, Nablus, 44839 Palestine; 5 Department of Pathology, An-Najah National University Hospital, Nablus, 44839 Palestine; 6 Department of Clinical and Community Pharmacy, College of Medicine and Health Sciences, An-Najah National University, Nablus, 44839 Palestine; 7 Poison Control and Drug Information Center (PCDIC), College of Medicine and Health Sciences, An-Najah National University, Nablus, 44839 Palestine; 8 Clinical Research Center, An-Najah National University Hospital, Nablus, 44839 Palestine

**Keywords:** Carbapenem resistant Enterobacterales, Resistance, Meropenem, *Escherichia coli*, *Klebsiella pneumoniae*

## Abstract

**Background:**

Carbapenem-resistant Enterobacterales (CREs) are a significant source of healthcare-associated infections. These bacteria are difficult to treat and have a high mortality rate due to high rates of antibiotic resistance. These pathogens are also linked to major outbreaks in healthcare institutions especially those with limited resources in infection prevention and control (IPC). Therefore, our study aimed to describe the epidemiology and clinical characteristics of patients with carbapenem-resistant Enterobacteriaceae in a referral hospital in a developing country.

**Methods:**

This was a retrospective cross-sectional study that included 218 patients admitted to An-Najah National University Hospital between January 1, 2021, and May 31, 2022. The target population was all patients with CRE infection or colonization in the hospital setting.

**Results:**

Of the 218 patients, 135 had CR-*Klebsiella pneumoniae* (61.9%), and 83 had CR-*Escherichia coli* (38.1%). Of these, 135 were male (61.9%) and 83 were female (38.1%), with a median age of 51 years (interquartile range 24–64). Malignancy was a common comorbidity in 36.7% of the patients. Approximately 18.3% of CRE patients were obtained from patients upon admission to the emergency department, the largest percentage among departments. Most CRE pathogens were isolated from rectal swabs, accounting for 61.3%. Among the 218 patients, colistin was the most widely used antimicrobial agent (13.3%). CR- *E. coli* showed resistance to amikacin in 23.8% of the pathogens tested and 85.7% for trimethoprim/sulfamethoxazole compared to CR- *K. pneumonia*, for which the resistance to trimethoprim/sulfamethoxazole was 74.1%, while for amikacin it was 64.2%. Regarding meropenem minimum inhibitory concentration, 85.7% of CR- *E. col*i were greater than 16 µg/mL compared to 84% of CR- *K. pneumonia* isolates.

**Conclusion:**

This study found that CRE is frequently reported in this tertiary care setting, implying the presence of selective pressure and transmission associated with healthcare setting. The antibiotics tested showed a variety of resistance rates, with CR-*K. pneumoniae* being more prevalent than CR-*E. coli*, and exhibiting an extremely high resistance pattern to the available therapeutic options.

## Introduction

Gram-negative infections that result from multidrug-resistant organisms (MDR) are usually associated with high mortality and morbidity and pose challenges to the healthcare system worldwide [1]. Gram-negative rods are one of the most common etiologic pathogens that cause ventilator-associated pneumonia in the United States of America [2]. Carbapenem-resistant Enterobacterales (CREs) are considered a serious cause of healthcare-associated infections, with ambiguity in their control [3]. As a result of the high level of antibiotic resistance in these pathogens, they are not easily treated and, therefore, are likely to cause high mortality rates. Some strains can be extensively transmitted through different modes, such as mobile genetic elements that enable the bacterium to produce carbapenemase enzymes. This could be related to the occurrence in healthcare settings along with the pertinent measures and resources of infection prevention and control (IPC) that are usually limited [3].

Well-established regimens, including colistin, aminoglycosides, tigecycline, and others, usually treat cases of infections resulting from resistance to carbapenems. Some of these agents are associated with concomitant side effects, including ototoxicity and nephrotoxicity. In addition, the emergence of pandrug-resistant organisms (PDR) has increased recently [4]. As an outcome measure, an annual death rate of more than 10 million as well as the probability of reaching costs of up to US$ 100 trillion by 2050 has been reported as a consequence of failing to control and prevent infections with multidrug-resistant organisms effectively [5].

According to the World Health Organization (WHO) Global Action Plan, one of the approaches to contain the spread and cross-transmission of resistant pathogens is to quickly generate and share epidemiological information specifically for each region [4].

This study aims to describe the epidemiology, demographic and clinical characteristics of patients, antimicrobial resistance of CRE isolates, and antibiotic utilization in treating these infections. To our knowledge, this is the first study investigating this type of microorganism in our community. It will help provide clinicians with the risk of colonization or infection with CRE and the best selection of empiric antimicrobials for treatment.

## Methods

### Study design

A retrospective cross-sectional study was conducted in a tertiary care teaching hospital with a capacity of 135 beds. We analyzed data collected from patients who were admitted to this hospital and showed the growth of CRE from different sites, either clinical samples or active surveillance tests, from January 1, 2021, to May 30, 2022. The required data were collected by reviewing the medical records for demographics and clinical characteristics and performing microbiology for antimicrobial susceptibility results.

### Setting

This study was conducted in Nablus, Palestine, in one of the main tertiary centers in Palestine, An-Najah National University Hospital, which serves over 300,000 people from all over the West Bank. This hospital has various departments, such as oncology, bone marrow transplantation, general surgery, cardiology, pediatrics and their relevant intensive care units (ICUs), including pediatric ICU, surgical ICU, medical ICU, and cardiac ICU.

### Population

All patients admitted to NNUH departments who were infected or colonized with CRE (*E. coli and/or K. pneumonia*) were included in the study. The collected data included 218 patients who showed CRE growth at any of these sites: rectum, nares, urine, blood, wound, tissue, fluid, sputum, and ear. The inclusion criteria were as follows: (1) all ages, (2) both sexes, (3) all hospital departments, and (4) all isolation sites, including clinical samples and active surveillance testing. Exclusion criteria: Other types of CRE pathogens were excluded as data for these microorganisms were unavailable. The study included all patients admitted to the hospital who exhibited the growth of CRE during the study time frame. After excluding 15 patients due to incomplete medical records, the final sample size was 218 patients, all of whom were infected or colonized with *E. coli*-CRE or *K. pneumoniae*-CRE between January 2021 and May 2022.

### Laboratory method of identification

Patients were considered CRE cases if they had any positive samples for CRE. However, patients with multiple CRE isolates on the same admission or from different specimen sites or dates within one month were counted only once, and the first positive CRE specimen was counted, according to CDC criteria [6]. Identification and antibiotic susceptibility Enterobacterales isolated from clinical samples supplied to the microbiology laboratory of An-Najah National University Hospital and identified as resistant to carbapenem using the automated Vitek 2 Compact system (bioMérieux, France) were considered. Susceptibility tests were performed by the Vitek 2 Compact (bioMérieux, France), and new colonies were used to make an inoculum in sterile saline to obtain a turbidity standard of 0.45–0.5 McFarland. AST-N204 cards were used to test for antimicrobial agents against aerobic gram-negative bacilli, including piperacillin ticarcillin-clavulanic acid, ticarcillin, piperacillin-tazobactam, amikacin, imipenem, tobramycin, meropenem, ceftazidime, gentamycin, nitrofurantoin, tobramycin, cefepime, ceftriaxone, cefotaxime, sulfamethoxazole-trimethoprim, ampicillin and ciprofloxacin, and the minimum inhibitory concentration (MIC) of each antibiotic was determined and evaluated using the Clinical and Laboratory Standards Institute (CLSI) M100-S30 document breakpoints [7, 8].

### Ethical considerations

All parts and aspects, including access to patient data, demographics, and clinical characteristics, were obtained and approved by the *Institutional Review Boards (IRB) of An-Najah National University*. Additionally, we ensured the confidentiality of patient data, as the data were only used for clinical research purposes and were not shared. Data were coded by numbers, not by patient names, and access was limited to study team members.

### Statistical analysis

We used Statistical Package for Social Sciences (IBM SPSS version 20) software to input and analyze the data. Descriptive statistics were employed to provide an overview of the data. For categorical variables, we reported frequencies and percentages, and for continuous variables, we calculated the mean and standard deviation.

## Results

### Demographic and clinical characteristics

During the study period, two hundred and eighteen patients were found, with a median age of ± interquartile range (IQR) of 51 (IQR, 24–64). More than half (61.9%) were men. Among these patients, 36.7% had malignancy as a primary disease, followed by hypertension and diabetes mellitus at 28.4% and 23.4%, respectively. Many patients have more than one comorbid disease. Regarding frequent hospital admissions as a risk factor for acquiring multidrug-resistant CRE, 35.3% of patients with CRE isolates had a history of three or more hospital admissions.

Based on the onset of CRE isolation in patients, they were categorized as healthcare-onset and community-onset. After applying these criteria, 122 patients (56%) showed CRE growth at admission as community-onset.

Of these 218 patients with CRE, 135 had CRE-*K. pneumoniae* (61.9%), and 83 had CRE-*E. coli* (38.1%). Regarding patient outcomes and crude mortality, 17.9% of the patients died during the admission course. All details can be found in Table 1. Positive CRE results were distributed among patients hospitalized in various departments, with admissions to the emergency department accounting for the highest percentage (18.3%) and admissions to the bone marrow transplant unit accounting for the lowest percentage (1.4%), as presented in Table 2.

**Table 1:** Demographic and clinical characteristics of patients with CRE

Variable	Frequency (%)
**Gender**	
Male	135 (61.9)
Female	83 (38.1)
Age (median, IQR)	51 (24–64)
**Comorbidities**	
Malignancy	80 (36.7)
Hypertension	62 (28.4)
Diabetes mellitus	51 (23.4)
Chronic kidney disease	19 (8.7)
Hypothyroidism	13 (6)
End stage renal disease	10 (4.6)
Liver cirrhosis	8 (3.6)
Congestive heart failure	7 (3.2)
Organ transplant	6 (2.7)
Inflammatory bowel disease	6 (2.7)
Rheumatoid arthritis	3 (1.4)
**Onset of infection**	
Present on admission	122 (56)
Hospital-onset	96 (44)
**History of three or more hospital admissions**	
No	141 (64.7)
Yes	77 (35.3)
**Organisms isolated**	
CR-*E. coli*	83 (38.1)
CR-*K. pneumonia*	135 (61.9)
**Outcome**	
Discharged	179 (82.1)
Died	39 (17.9)
Total	218 (100)

**Table 2:** Wards of patients with CRE

Ward	N (%)
Emergency	40 (18.3)
General surgery	37 (17)
Surgical ICU	33 (15.1)
Medical ICU	21 (9.6)
Pediatrics	19 (8.7)
Vascular surgery	15 (6.9)
Cardiac surgery	14 (6.4)
Internal medicine	13 (6)
Oncology	9 (4.1)
Pediatric ICU	9 (4.1)
Cardiac care unit	5 (2.3)
Bone marrow transplant unit	3 (1.4)
Total	218 (100)

### Isolation site and antibiotic utilization

Regarding isolation sites, most of the CRE isolates were obtained from rectal swabs as active surveillance tests with 61.3% of the tested samples, followed by urine cultures with 12.7%. Blood cultures with CRE accounted for 7.3%, as illustrated in Table 3.

**Table 3:** Sources of CRE isolated from patients

Source	N (%)
Rectal swab	184 (61.3)
Urine culture	38 (12.7)
Wound/tissue culture	22 (7.3)
Blood culture	16 (5.3)
Sputum culture	16 (5.3)
Nasal swab	12 (4)
Fluid culture	11 (3.7)
Ear culture	1 (0.3)

As there are limited options for CRE treatment, especially in low-income countries, the vast majority of antibiotics are used in combination regimens. Among those who were treated with CRE isolated from clinical samples, the antimicrobial most utilized was colistin (26.2%), followed by amikacin (19.6%) and tigecycline (9.3%), as shown in Table 4.

**Table 4:** Antibiotics used for patients with CRE

Antibiotic	N (%)
Colistin	28 (26.2)
Amikacin	21 (19.6)
Piperacillin-Tazobactam	13 (12.1)
Tigecycline	10 (9.3)
Meropenem	9 (8.4)
Gentamicin	6 (5.6)
Trimethoprim-Sulfamethoxazole	4 (3.7)

### Antibiotic sensitivity of CRE isolated during the study period

CRE is resistant to cephalosporins and most beta-lactams, which we found in the microbiology profile of the cultures. CRE-*E. coli* showed resistance to amikacin in 23.8% of isolates and, trimethoprim/sulfamethoxazole in 85.7%, and 66.7% of the CRE-E. coli isolates were resistant to gentamicin. For CRE in urine, the sensitivity for fosfomycin and nitrofurantoin was 75.1% and 25%, respectively.

Meanwhile, for CR-*K. pneumoniae*, resistance rates were 90.1% for ciprofloxacin, 93.8% for cefepime and 92.9% for nitrofurantoin. The resistance rate for trimethoprim/sulfamethoxazole was 74.1%, while for amikacin, it was 64.2% compared with gentamicin, which had the lowest resistance rate 46.9%. The antibiotic resistance of the CRE isolates is listed in Table 5.

**Table 5:** Antibiotic resistance of CRE isolated from clinical samples during the study period

	Ampicillin	Amoxicillin-clavulanate	Piperacillin	Cefotaxime	Ceftazidime	Ceftriaxone	Cefepime	Ertapenem	Imipenem	Meropenem	Amikacin	Gentamicin	Ciprofloxacin	Nitrofurantoin	Fosfomycin	Norfloxacin	Trimethoprim/ sulfamethoxazole
CR-*E. coli* tested isolates for antibiotic resistance	21	21	21	21	21	8	19	21	21	21	21	21	21	8	7	8	21
Resistant isolates, (%resistance)	21 (100)	21 (100)	21 (100)	21 (100)	21 (100)	7 (87.5)	18 (94.7)	21 (100)	19 (90.5)	20 (95.2)	5 (23.8)	14 (66.7)	19 (90.5)	2 (25)	4 (57.1)	7 (87.5)	18 (85.7)
CR*-K. pneumoniae* tested isolates for ntibiotic resistance	81	81	81	81	81	8	80	77	81	81	81	81	81	28	23	30	81
Resistant isolates, (%resistance)	81 (100)	80 (98.8)	80 (98.8)	80 (98.8)	80 (98.8)	7 (87.5)	75 (93.8)	77 (100)	81 (100)	81 (100)	52 (64.2)	38 (46.9)	73 (90.1)	26 (92.9)	14 (60.9)	25 (83.3)	60 (74.1)

### Minimum inhibitory concentration (MIC) (µg/mL) of meropenem for CRE isolates

Among the isolated samples, with respect to the meropenem MIC, 85.7% of CR- *E. coli* were above the MIC of 16 µg/mL, and 84% of the CR- *K. pneumoni*a isolates had a MIC of 16 µg/mL or more, as shown in Table 6.

**Table 6:** Minimum inhibitory concentration (MIC) (µg/mL) of meropenem for CRE isolated during the study period

	CR-*E. coli* (21)	CR*-K. pneumoniae* (81)	Total (102)
	n (%)	n (%)	n (%)
MIC < 16	3 (14.3)	13 (16)	16 (15.7)
MIC ≥ 16	18 (85.7)	68 (84)	86 (84.3)

## Discussion

Carbapenem-resistant Enterobacterales (CREs) are a dominant threat to patient health in healthcare settings worldwide. CRE strains consistently have limited therapeutic options, often associated with increased toxicities, and require prolonged treatment, leading to a higher cost burden than carbapenem-susceptible strains [9]. In this study, 61.9% of carbapenem-resistant isolates were *K. pneumoniae.* Compared to each other, 38.1% were *E. coli*, according to a study by Chotiprasitsaku et al. that showed that 70.7% of the isolated CRE were *K. pneumoniae* [10].

Furthermore, the results of a study that was conducted to evaluate the importance of performing active surveillance tests for CRE showed that the most commonly isolated strains were *K. pneumoniae* (83.16%), followed by *Enterobacter cloacae* (9.76%) and *E. coli* (4.38%) [11]. Furthermore, *K. pneumonia* was predominant in 25.4% of the isolates compared to 18.4% of *E. coli* [12].

The majority of patients who showed CRE-positive specimens were males (n = 13, 61.9%), with a median age of 51 years (IQR 24–64). These results are somewhat similar to those of Gomides et al., who found that the average age of CRE cases was 52.42 ± 19.34 years (range 13–97 years), and males were more predominant (65.31%) than females (1.88:1), with a higher discharge rate in our study (82.1%) than in this study (5.3%) [11].

The comorbidities of patients with CRE isolates varied. Among them, malignancy was the most common (n = 80, 36.7%), which is consistent with a study conducted in India that showed a high prevalence of CRE among cancer patients [13]. Other reported comorbid diseases in our study were hypertension (n = 62, 28.4%), diabetes mellitus (n = 51, 23.4%), chronic kidney disease (n = 19, 8.7%), and other comorbidities, such as hypothyroidism, end-stage renal disease, liver cirrhosis, heart failure, organ transplants, inflammatory bowel disease, and rheumatoid arthritis.

In our study, the onset of CRE acquisition was also investigated. 56% of the patients had CRE-positive samples in the first three days of admission, which is considered present on admission and may be attributed to other hospital visits, as the hospital setting of the study setting hospital is a tertiary health care institution that receives referrals from other hospitals in the West Bank and Gaza.

In addition, 36.7% of the patients were cancer patients with recurrent chemotherapy treatment visits. In children with malignancy, 56% of *E. coli* and 37% of isolated *K. pneumoniae* were CRE [14].

Due to the limited availability of treatment options, infections caused by CRE are often associated with high rates of morbidity and mortality. We studied patient outcomes in terms of crude mortality, defined as in-hospital death within 30 days of a CRE positive result. The 30-day all-cause mortality after CRE positivity was found to be 17.9%, which is consistent with a study conducted in China in which mortality among patients was 17.2% [15], while a study investigating 60-day all-cause mortality in a tertiary care hospital in Cuba resulted in a mortality rate [16]. However, the results of this investigation as a case series of CRE infections between 2011 and 2014 in Lebanon showed a mortality rate of 27.5% in the hospital, which was higher than our findings [17]. Several variables, including the primary site of infection and antibiotic use 24 h before CRE infection, were potentially associated with mortality. Additionally, the mortality group experienced many comorbidities, such as sepsis, unsuccessful treatments, and respiratory failure.

Active surveillance testing (AST) to detect CRE colonized patients for proper patient isolation purposes and, in some cases, empiric antibiotic selection is applied in this hospital as a facility-specific policy in which patients with a high risk of MDRO are eligible for the criteria of AST (i.e., referred from another hospital, admitted to any hospital for more than 48 h in the last six months, previous history of MDRO, upon admission to the ICU, then weekly, rectal swabs are repeated or as required by the infection prevention and control team). As part of AST, rectal swabs represented most CRE isolates. The finding of a high prevalence of CRE isolation from rectal swabs was consistent with a study conducted at a university hospital that rectal concluded that CRE colonization is very common in high-risk patients from the ICU and hematopoietic stem cell transplantation (HSCT) departments and predominant colonization of carbapenem-resistant *K. pneumonia* [18]. Understanding colonization as a critical step in infection progression provides a reason to detect colonized patients and potentially design intervention measures to avoid subsequent infection [19]. Approximately 40.6% of the CRE-infected patients in our study were colonized with CRE prior to developing infection compared to the 65% rate in the study by Xia Chen et al. [20]. Moreover, some data suggest that active surveillance tests performed on admission may play a role in preventing the spread of resistant gram-negative organisms in healthcare institutions with regard to widespread cross-transmission and outbreaks [21].

Regarding clinical specimens, urine was the most common source of CRE isolates (n = 38, 12.7%), which was consistent with the findings of Daniel et al. (31.8%) [22] and Moghnieh et al. (31% of cases were found in urine) [23]. In a pediatric study that described the demographics and clinical data of patients under one year who showed CRE growth, *K. pneumonia* was predominant (60.4%), and sputum (37.5%) was the most common clinical sample compared to urinary cultures (25%) [24]. However, in a Tunisian study evaluating the occurrence and characterization of CRE pathogens, the highest CRE rate was isolated from blood samples (28%), followed by anal swabs (21.5%) and urine samples (18.4%) [25]. The discrepancy in rates may be attributed to the early detection of CRE pathogens in the studied hospital through AST and early implementation of infection prevention measures that help prevent the spread of pathogens to sterile body sites and the development of infection.

Associated healthcare risk factors that improve CRE infection include prolonged hospitalization, the existence of invasive devices, attendance in high-risk units such as an ICU, and previous exposure to broad-spectrum antibiotics [26]. Our study identified some possible risk factors that other investigators highlighted [27]. We found that 68 cases of CRE were isolated from ICU patients, which comprises an overall prevalence of 31.1% of all CRE cases. This may be explained by the longer hospital stay in this category and greater utilization of medical devices and broad-spectrum antibiotics, exposing them to greater risk than patients in other departments. A study carried out in Gaza (a nearby area) revealed that the ICUs had the highest resistance rate of Enterobacteriaceae to carbapenem, with 52.9% of all isolated Enterobacteriaceae [28]. In Morocco, Delaguio et al. found that most of CRE was isolated from the neonatal unit (14%), followed by the departments of urology-nephrology (11%) and plastic surgery (10%) [29].

Gram-negative bacteria, particularly CREs, are among the world’s most significant public health problems as a result of their extensive antibiotic resistance. Only last-line antibiotics such as colistin, fosfomycin, and tigecycline were effective against most of these isolates [30, 31].

Patients received multiple antimicrobial therapies according to clinical culture and sensitivity results; therefore, patients who were colonized with CRE in the form of nasal or rectal swabs did not receive antibiotics, as they did not have signs and symptoms of infection. The swabs were performed as part of active surveillance and isolation purposes. Therefore, not all samples were tested for all antibiotics, as active surveillance samples are excluded from sensitivity testing according to microbiology and CLSI guidelines [8]. For CRE strains in colonized patients, Lin et al. showed that compared to *K. pneumonia*, *E. coli* was more susceptible to gentamicin (59.3% vs. 21.1%) and amikacin (87.0% vs. 45.1%) [32]. For therapeutic purposes in our study and according to the results guidelines and observational studies for the management of CRE infection, patients with CRE were managed with a combined regimen [31, 33]. It was shown that the most widely used antibiotic among our patients with CRE was colistin (13.3%), which is still considered a viable and key treatment option for CRE infections [34]; colistin was also found to be the cornerstone for CRE management in other studies [16]. Other treatment options offered to patients were amikacin (9.6%) and tigecycline (4.6%). Meanwhile, meropenem was administered as a high-dose and extended infusion protocol in 4.1% and gentamycin in 2.7% of the regimens. On the other hand, a study conducted in Dammam showed that 37% of the patients were treated with tigecycline as a targeted therapy, followed by colistin 28%, amikacin 21%, and gentamicin 11% (16).

Previously, aminoglycosides were highlighted as the main line in CRE treatment, since they could be the only antimicrobials to which CRE isolates showed in vitro sensitivity [35, 36]. However, high resistance rates to aminoglycosides have been reported in some studies in which only 20.4% of the CRE isolates were gentamicin susceptible [37]. Another study found that only 10.4% and 13.0% of CRE pathogens were susceptible to amikacin and gentamicin, respectively [38]. Furthermore, Wu et al. reported an amikacin resistance rate of 29% and a gentamycin resistance rate of 76% for isolated CRE [39]. However, our study showed higher susceptibility of CRE to amikacin and gentamicin, with differences between *E. coli* and *K. pneumonia* (76.2% and 33.6% for CR*- E. coli* and 35.1% and 53.1% for *K. pneumonia*, respectively). Concerning ciprofloxacin and trimethoprim-sulfamethoxazole (94%), a Bahraini study showed that 79% of all CRE isolates were resistant to trimethoprim-sulfamethoxazole, and 94% of the isolates tested were ciprofloxacin resistant [40]. This was in agreement with our study, which showed that 85.7% of the *E. coli* isolates were resistant to trimethoprim-sulfamethoxazole, while 74.1% of the *K. pneumonia isolates* showed resistance to trimethoprim-sulfamethoxazole. For ciprofloxacin, the highest resistance rate was observed with *E. coli* (66.7%), which was lower than that observed in an earlier Bahraini study [40] and then the rate reported in a Chinese study (95% for *K. pneumoniae* and 88% for *E. coli*) [39].

The minimum inhibitory concentration (MIC) of an antibiotic is defined as the lowest needed concentration of that antibiotic to prevent visible growth of bacteria or bacteria. Regarding the interpretation of meropenem MIC for *E. coli* and *K. pneumonia* and according to the CLSI breakpoints, when MIC exceeds 4 µg/mL, the bacteria are considered resistant to carbapenems [41]. Among the isolated samples, 85.7% of CR-*E. coli* had a MIC of more than 16 µg/mL, while 84.3% of the samples of CR-*K. pneumonia* showed a MIC of more than 16 µg/mL. A study on CRE in children with cancer found that all isolates were resistant to carbapenem, with a MIC < 4–8 µg/mL in 100 (45%) and > 8 µg/mL in 153 (55%) [14]. Previously, it was common practice to treat CRE-causing infections with high meropenem MICs (8–16 mcg / ml) using extended-infusion meropenem in combination with another drug, often polymyxin or aminoglycosides [34]. However, subsequent observational and RCT data revealed that these regimens were associated with higher rates of mortality and nephrotoxicity compared to newer β-lactam-β-lactamase inhibitor agents to treat CRE infections. As a result, the IDSA panel’s most recent guidelines do not recommend the use of extended infusion carbapenems, with or without a second drug, to treat CRE when meropenem non-susceptibility is confirmed [42, 43].

Overuse and inappropriate use of broad-spectrum antimicrobials, which aid in the development of various resistance mechanisms by these pathogens, along with the lack of effective antibiotic stewardship programs, have helped hasten the cycle of emerging resistance; that is, the lack of well-established infection prevention and control practices are all factors that have contributed to the persistence and spread [44]. The increasing incidence of infections caused by Gram-negative bacteria from MDR creates substantial difficulty in optimal empirical antibiotic selection for critically ill patients [44]. Containing the spread of MDR gram-negative bacilli, predominantly CRE, is challenging, and the adoption of multimodal infection control care bundles is vital to prevent outbreaks and catastrophic sequela [45].

### Strengths and limitations

Although this paper is the first in Palestine to study the topic of CRE isolates, our study has several limitations. First, it is a retrospective descriptive study in which data were collected from a single center that studied only two carbapenems-resistant species; thus, it may not be representative of other centers. Second, it did not assess the change in antibiotic resistance throughout the year or year over year. Finally, due to limited resources in developing countries, molecular testing for CRE is unavailable at our institution, and the results of colistin sensitivity were not feasible to collect during the study period.

## Conclusions

This study found that CRE is frequently reported and spreads in the center of study. More than half of the cases showed CRE growth either as colonization or infection at the time of admission, and CR-*K. pneumoniae* was more prevalent than CR-*E. coli*, with an extremely high resistance pattern to available therapeutic options.

### Clinical perspectives and recommendations

- CRE represent a major public health risk, especially in immunosuppressed people. A referral hospital in a developing country found that CR-K. *pneumoniae* causes most health care infections. This suggests improved surveillance and control to stop the spread of antibiotic-resistant microorganisms.
- Healthy environments must limit CRE transmission through hand hygiene, contact protection, and environmental cleaning. Healthcare professionals should also learn about the use of antibiotics, antimicrobial management, and stewardship to prevent resistant strains.
- It is recommended to prioritize the screening and isolation of patients at a higher risk, such as individuals with a history of CRE infection, recent hospitalization, or exposure to healthcare institutions outside their country of residence. Furthermore, the implementation of active surveillance programs can effectively facilitate the identification of colonized patients and mitigate the risk of transmission.
- The study suggests using amikacin and trimethoprim/sulfamethoxazole, which have shown some efficacy against CR- *E. coli*, to improve treatment outcomes. However, regional resistance patterns can affect the efficacy of certain antibiotics, requiring sensitivity tests to guide treatment.
- Modify antimicrobial testing and susceptibility panels to provide direct susceptibility results of the available treatment options, such as colistin and tigecycline.

## Data Availability

Data and materials used in this work are available from the corresponding authors upon request.

## Abbreviations

AST: Active surveillance testing
CRE: Carbapenem-resistant Enterobacterales
HSCT: Hematopoietic stem cell transplantation
ICUs: Intensive care units
IDSA: Infectious Disease Society of America
IPC: Infection prevention and control
IQR: Interquartile range
IRB: Institutional Review Board
MDR: Multidrug resistant
MIC: Minimum inhibitory concentration
PDR: Pandrug resistant
